# Hyperglycemia Induces Altered Expressions of Angiogenesis Associated Molecules in the Trophoblast

**DOI:** 10.1155/2013/457971

**Published:** 2013-07-25

**Authors:** Shu-Chun Chang, Wei-Chung Vivian Yang

**Affiliations:** ^1^Graduate Institute of Biomedical Materials and Tissue Engineering, College of Oral Medicine, Taipei Medical University, 250 Wu-Hsin Street, Taipei 110, Taiwan; ^2^The Ph.D. Program for Translational Medicine, College of Medical Science and Technology, Taipei Medical University and Academia Sinica, 250 Wu-Hsin Street, Taipei 110, Taiwan; ^3^Center for Reproductive Medicine, Taipei Medical University Hospital, 252 Wu-Hsin Street, Taipei 110, Taiwan; ^4^Center for Translational Medicine, Taipei Medical University, 250 Wu-Hsin Street, Taipei 110, Taiwan

## Abstract

We previously reported that the increased level of perlecan with altered glycosaminoglycan (GAG) substitution was present in the placenta with gestational diabetes mellitus (GDM) and in the trophoblasts cultured under hyperglycemic condition. Trophoblast is the first cell lineage to differentiate, invasive, and migrate into the vessel tissues of placenta and fetal membrane during pregnancy. Therefore, active matrix remodeling and vessel formation must occur during placentation. In this study, we further investigated whether hyperglycemia-induced alterations of perlecan in the extracellular matrix (ECM) affect the proliferation and the expressions of angiogenesis-related growth factors and cytokines in the trophoblasts. 3A-Sub-E trophoblastic cells cultured in high glucose medium were conducted to mimic the hyperglycemic condition. Results showed that the hyperglycemia-induced GAG alterations in the cell surface perlecan as well as in the ECM indeed upregulated the expressions of IL-6, IL-8, and MCP-1 and the activities of MMP-2 and MMP-9 and downregulated the expressions of TIMP-2. A regulatory molecular mechanism of hyperglycemia-induced alterations of the cell surface proteoglycans and the ECM remodeling on the expressions of angiogenesis-related cytokines and growth factors in trophoblasts was proposed. This mechanism may contribute to the aberrant placental structure and the maternal and fetal complications during development.

## 1. Introduction

Placental development is important for fetal health. Maternal diabetes or gestational diabetes mellitus (GDM) induced hyperglycemia could cause placental development abnormality that might result in maternal complications and poor fetal outcomes [1, 2]. Perlecan, a heparin sulfate proteoglycan, is a major component of basement membrane and is involved in blood vessel formation by regulation of cell proliferation, growth factors, and cytokines in the extracellular matrix [3–5]. In addition, perlecan can bind proangiogenic growth factors such as fibroblast growth factors (FGFs) and vascular endothelial growth factor (VEGF) and present them to their receptors on the cell surface [3, 4]. During embryonic development, perlecan is located in the apical surface of trophectoderm functioning in the initial blastocyst-uterine epithelium interaction for embryo preimplantation [6]. It appears that the trophoblast involved embryo implantation is mediated by heparin or heparin sulfate binding protein on uterine epithelium [7–9]. We previously have shown that perlecan is mainly expressed in the trophoblast and vessel basement membranes, and both the protein and mRNA levels of placental perlecan were significantly increased in the third trimester placentas with gestational diabetes mellitus (GDM) as well as in trophoblast cells cultured at high glucose (30 mM) condition [10]. We have also demonstrated that induced hyperglycemic condition increased chondroitin sulfate substitution on placental perlecan and in the cultured trophoblasts [11], suggesting that induced hyperglycemia altered perlecan expression may contribute to the abnormality of placental development and the maternal and fetal complications.

Trophoblast is the first cell lineage to differentiate, invasive, and migrate into the vessel tissues of placenta and fetal membrane during pregnancy [12]. Growth factors, cytokines, and angiogenic molecules were found to regulate trophoblast motility [13]. In this study, the effect of hyperglycemia on growth factors, cytokines and angiogenic molecules that may regulate trophoblast migration was studied. In addition, whether any of the induced hyperglycemia altered expressions of cytokines and angiogenic molecules were mediated by the altered perlecan expression was also investigated. 

## 2. Materials and Methods

### 2.1. Cell Culture

The trophoblast cell line 3A-Sub-E (ATCC CRL-1584) was cultured in MEM (Gibco), containing 10% FBS (Gibco), 100 unit/mL penicillin, and 100 *μ*g/mL streptomycin (Gibco). For the hyperglycemia mimicking condition cell culture, the cells were cultured in the MEM medium with 1% FBS supplemented with 5.6 mmol/L D-glucose (the normal glycemic control), 30 mmol/L D-glucose (Merck) (the hyperglycemic group), or with 5.6 mmol/L D-glucose and 24.4 mmol/L mannitol (Sigma) (the osmotic matched hyperglycemic control). Trypan blue exclusion test was carried out to determine the number of viable cells. 

### 2.2. Isolation of Proteoglycans in 3A-Sub-E Cells

The protocol for isolation of proteoglycans from cells is followed based on the report by Fischer et al. [14] with some modifications. 3A-Sub-E cells cultured in hyperglycemic, normal glycemic, and the osmotic control medium were harvested for the isolation of proteoglycans on 24 h, 48 h, and 72 h posttreatment. The conditioned medium was discarded, and the cells were washed with PBS. Subsequently, 4 M guanidine HCl in 50 mM sodium acetate, pH 5.8 with 0.1% CHAPS, and protease inhibitor cocktail for mammalian cells in appropriate amount (Sigma) were added into the cells and incubated on an orbital shaker at 4°C for overnight. The cell debris was scraped, and the extract solution was collected and dialyzed to 7 M Urea in 50 mM Tris, pH 6.8 with 150 mM NaCl, and protease inhibitor cocktail at 4°C for overnight. The supernatant containing proteoglycans was obtained by centrifugation at 12,000 rpm for 20 min and stored at −20°C for further analysis.

### 2.3. Immunoprecipitation of Perlecan

To reduce the nonspecific protein contamination, a two-cycle immunoprecipitation procedure was carried out for the study [15]. The cell extract (250–370 *μ*g) was preincubated with the protein G resin (Sigma), 50% in 10 mM Tris, pH 8.0 (100 *μ*L for the cell extract for each reaction) at 4°C for 1 h to remove any nonspecific protein binding to the protein G. Followed by centrifugation at 800 g for 20 s, the precleared protein supernatant was transferred to a clean tube for another immunoprecipitation by the addition of the monoclonal antibody against human perlecan, clone A7L6 (Chemicon), 0.5 *μ*L together with new 50% protein G resin slurry in 10 mM Tris, pH 8.0 for end-over-end incubation at 4°C for overnight. The sample was then centrifuged at 800 g for 20 sec, and the supernatant was removed. Subsequently, the pellet was washed with 1 mL ice cold 10 mM Tris, pH 8. After three washes, the absorbed perlecan was eluted by the addition of SDS containing sample buffer and boiled for 5 min before subjected to SDS-6% polyacrylamide gel electrophoresis (PAGE) for analysis.

### 2.4. Sodium Dodecyl Sulfate-Polyacrylamide Gel Electrophoresis of Proteoglycans

The immunoprecipitated perlecan was analyzed by electrophoresis on a SDS-6% polyacrylamide gel. After fixation with 40% ethanol and 10% acetic acid, the gel was stained with alcian blue solution (0.5% alcian blue in 3% acetic acid) and Coomassie blue G25 for the observations of glycan chains and the core protein, respectively.

### 2.5. Antibody Arrays Analysis for Angiogenesis Associated Molecules

Both cell lysates and the conditioned media were collected from 3A-Sub-E cells cultured under hyperglycemic, the osmotic matched high glucose control, and normal glycemic conditions. Human angiogenesis array (RayBiotech) consisted of 20 different angiogenesis-related antibodies spotted in duplicates onto a membrane was used for the analysis. The membranes were incubated with 1X Blocking Buffer (10% bovine serum albumin in Tris-buffered saline) at room temperature for 1 h. One milliliter of conditioned medium or cell lysates was added to each membrane in separate wells of a 6-well plate. The membranes were then shaken at 110 rpm at room temperature for 2 h. Followed by the washes with Wash Buffer I and II in another new 6-well plate, 1 mL of a 1 : 250 dilution of the biotin-conjugated antibodies was added to each membrane, respectively, and the mixture was incubated on a shaker for 2 h at room temperature. Following by the washes, the membranes were incubated with horseradish peroxidase (HRP-) conjugated streptavidin (1 : 1,000 dilution) for 1 h at room temperature. After a thorough wash, the membranes were exposed to a peroxidase substrate for 5 min in the dark before imaging. Two or four individual membranes were placed side by side in a plastic protective folder and sealed. Imaging was done using an imaging system. Exposure times ranged from 3 min to overnight. All target signals from antibody array were quantified by Scion Image software. Horseradish peroxidase (HRP-) conjugated antibody at 6 spots served as a positive control and was also used to identify the membrane orientation. For each spot, the net density of gray level was determined by subtracting the background from the total raw density of gray levels. The experiment was repeated to confirm the reproducibility. 

### 2.6. GAG Degradation Treatment

To release heparin/heparin sulfate, appropriate amount of heparinase III (HIII) from *Flavobacterium heparinum* (Sigma) in 10 mM Tris (pH 8.0) containing 0.1 mg/mL BSA and 4 mM CaCl_2_ was added at 25°C for 3 h. For chondroitin sulfate degradation, chondroitinase ABC (Chabc) from *Proteus vulgaris* (Sigma) in 10 mM Tris (pH 8.0), 60 mM sodium acetate, and 0.02% BSA was used for the incubation at 37°C for 1 h. For degradation of both heparin/heparin sulfate and chondroitin sulfate, the samples were incubated with heparanase III prior to chondroitinase ABC.

### 2.7. Real-Time Quantitative Polymerase Chain Reaction (RT-qPCR) Analysis

Total RNA was extracted using TRIzol reagent (Ambion Life Technologies). One microgram of total RNA was used to perform reversed transcriptase-polymerase chain reaction (RT-PCR) using QuantiTect Reverse Transcription kit (Qiagen). 100 ng of reverse-transcribed cDNA per sample with desired primers for the targeted gene (Table 1) was used to perform real-time PCR using a Rotor-Gene Q (Qiagen). The quantitation was performed as absolute number of DNA copies per sample using QuantiFast SYBR Green PCR Kit (Qiagen) and its software (Rotor-Gene Q Series Softwares version 2.1.0). The amount of transcripts was normalized to that of *β*-actin. The results are presented as relative values as ratio of the number of copies for the targeted gene and *β*-actin. Each gene was analyzed in duplicates. Three independent experiments were carried out for data validation. 

### 2.8. Zymography Analysis for MMP Activity

Equal amount of the total protein in the cultured conditional medium from 3A-Sub-E cells with high glucose (30 mM) and normal glucose (5.8 mM) followed by the treatment with the indicated GAG degradation enzyme was collected, respectively, and separated on a 10% gelatin zymography gel (Life Science). The enzyme activity was performed based upon the instruction by the manufacture. The gel image was developed by ChemiDoc imager (BioRad). 

## 3. Results and Discussions

### 3.1. Hyperglycemic Condition Has No Effect on the Cell Proliferation of Trophoblast 3A-Sub-E Cells in Short-Term Culture

**Figure 1:**
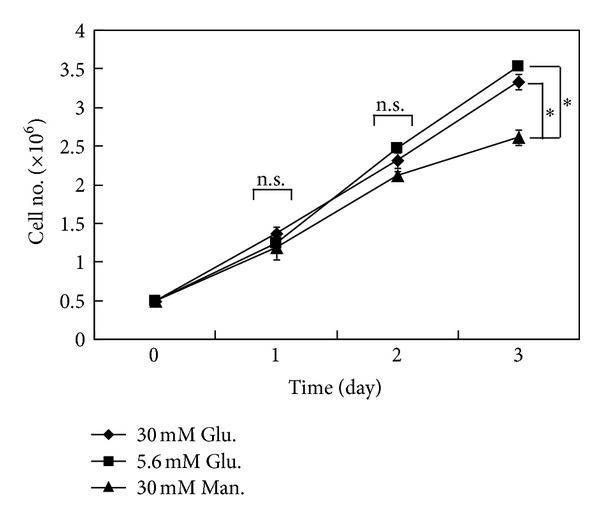
The effect of hyperglycemic condition on the proliferation of trophoblasts. Trypan blue exclusion test was carried out to determine the number of viable cells in the suspension harvested from 30 mmol/L D-glucose (30 mM Glu., the hyperglycemic group), 5.6 mmol/L D-glucose (5.6 mM Glu., the normal glycemic control), or 5.6 mmol/L D-glucose with 24.4 mmol/L mannitol (30 mM Man., the osmotic control) culture for 24 h, 48 h, and 72 h (1–3 days). Data are presented as mean ± SD (*n* = 3). *P < 0.05. n.s., not significant.

Hyperglycemia may affect the cell proliferation. The number of viable trophoblast 3A-Sub-E cells cultured under hyperglycemic (30 mmol/L D-glucose), normal glycemic control (5.6 mmol/L D-glucose), or the osmotic control (5.6 mmol/L D-glucose with 24.4 mmol/L mannitol) conditions were followed for 3 days. No significant difference on the proliferation of the cells cultured under hyperglycemic, normal, or the osmotic control for 48 h (Figure 1). This finding was similar to a first trimester trophoblast-derived cell line, ACH-3P under hyperglycemic treatment (25 mmol/L D(+)-glucose); there was no effect on the cell proliferation up to 3 days of the culture [16]. However, hyperglycemic condition might impair the proliferation of endothelial cells for short term culture (1–3 days) [17] and enhanced the proliferation for long-term culture [18]. In addition, glucose also stimulated smooth muscle cell proliferation [19]. These results suggest that hyperglycemic condition has less effect on trophoblast proliferation. Glucose consumption may vary depending on the cell type and the cultured conditions [20].

### 3.2. The Effect of Hyperglycemia on the Expression of Cell-Associated Perlecan in Trophoblast 3A-Sub-E Cells

**Figure 2:**
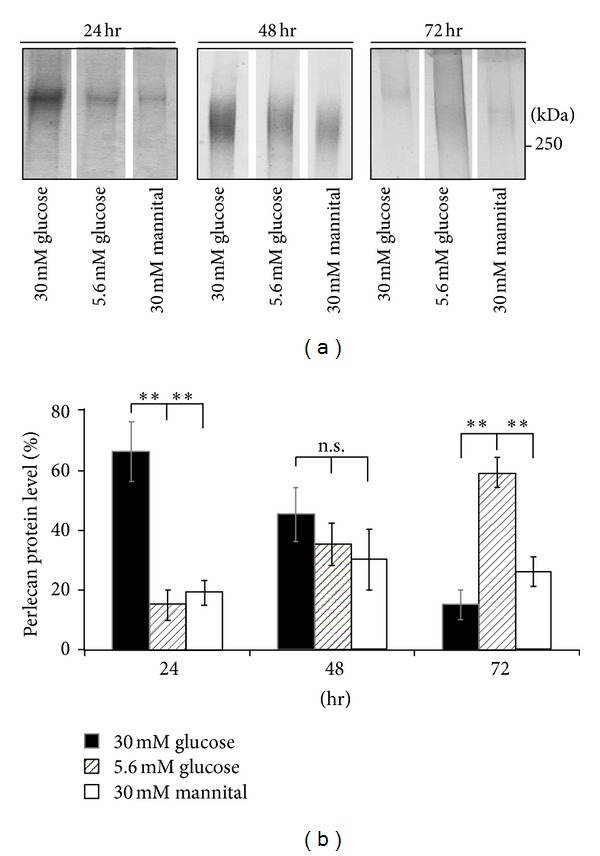
The expression of cell-associated perlecan in trophoblasts upon treatment with high-glucose condition. To characterize perlecan expression profile upon treatment with high-glucose condition, time course analysis of perlecan immunoprecipitation was conducted in 3A-Sub-E cell lysates with pretreatment of 30 mM D-glucose for 24, 48, and 72 h. (a) Perlecan expression was electrophoresis and shown on a 6% SDS polyacrylamide gel stained with alcian blue and Coomassie blue G25 solutions. (b) Quantitative results of perlecan protein from cell lysates under different stimulations. The expression of perlecan was performed as mean ± SD by quantification of the relative intensity of the scanned smear bands from double stains with Coomassie blue and alcian blue (*n* = 3). The total intensity (30 mM Glu. + 5.6 mM Glu. + 30 mM Man.) from each time point was considered as 100. **P < 0.01. n.s., not significant.

**Figure 3:**
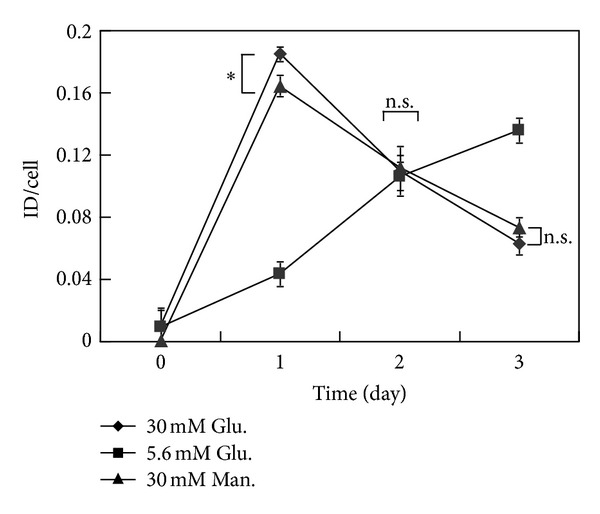
Altered cell-associated perlecan expression upon stimulation with high glucose and high osmotic condition in proliferated trophoblasts. The cell-associated perlecan expression according to the proliferation of 3A-Sub-E cells under high and normal glucose treatment was analyzed. It appeared that the cell-associated perlecan expression is increased 24 h posttreatment with either hyperglycemic condition (30 mM Glu.) or the osmotic matched control (30 mM Man.) and gradually decreased 24–72 h posttreatment. The hyperglycemia mimicking condition (30 mM Glu.) indeed upregulated the level of perlecan expression on 24 h posttreatment compared to that in the osmotic control (30 mM Man.). The cell-associated perlecan expression under normal glycemic condition (5.6 mM Glu.) was gradually increased as the cell proliferated. Data are presented as mean ± SD (*n* = 3). *P < 0.05. **P < 0.01. n.s., not significant.

Our previous studies reported that the expression of perlecan was increased in the third trimester placenta with gestational diabetes mellitus (GDM), and histology studies revealed that perlecan was mainly expressed around trophoblasts; in addition, the GDM placental perlecan had increased chondroitin sulfate content on its GAG chains [10]. Since perlecan plays important roles on cytotrophoblast and uterin cell interface interaction, in this study, we further investigated the cell-association activity of perlecan in trophoblasts upon treatment with high-glucose condition. A time course study for monitoring the cell associated perlecan by immunoprecipitation was conducted in 3A-Sub-E cell lysates.  Figure 2 showed that the cell-associated perlecan was gradually decreased upon treatment with high-glucose from 24 h to 72 h whereas the cell-associated perlecan was gradually increased in normal glycemic control cells (Figure 3). It suggested that hyperglycemic condition induced more perlecan deposited on the cell surface and osmotic effect might contribute at least in part on the increased density of perlecan present on the cell surface. 

### 3.3. The Effect of Hyperglycemia on the Expression of Angiogenesis Associated Proinflammatory Cytokines, C-C Motif Chemokines, and Matrix Metalloproteinase Inhibitors

**Figure 4:**
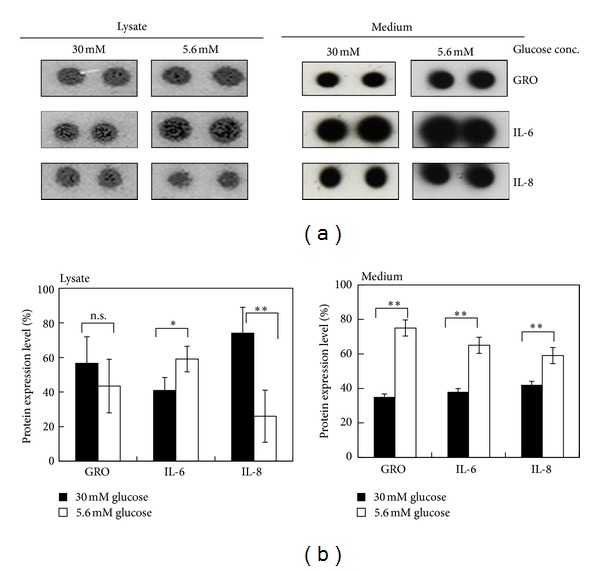
The effect of hyperglycemia on the expression of proinflammatory cytokines in 3A-Sub-E trophoblast cells. 3A-Sub-E cells were exposed to 30 mM D-glucose or to 5.6 mM of D-glucose (control) for 48 h. Cell lysates (250–370 *μ*g) and the cultured conditioned media were collected for the antibody array analysis, respectively. (a) Representative dot image of the indicated proinflammatory cytokines. (b) Average intensity for each pair of cytokine spots was quantitatively measured by Scion Image software. Data are presented as mean ± SD (*n* = 4). Each cytokine is represented by duplicate spots as GRO (growth-regulated oncogene), IL-6 (interleukin-6), IL-8 (interleukin-8), and control (HRP-conjugated antibody as a positive control). *P < 0.05. **P < 0.01. n.s., not significant.

Subsequently, we used high glucose treatment between 24 h and 48 h in 3A-Sub-E cells to study the effect of hyperglycemia on perlecan, cytokines, and the angiogenesis associated molecule expressions. Antibody array analysis showed that the proinflammatory cytokine, growth-regulated oncogene (GRO), interleukin-8 (IL-8), and interleukin-6 (IL-6) were expressed in 3A-Sub-E cells (Figure 4(a)). Hyperglycemic condition did not affect the expression of IL6 whereas significant expression of IL-8 was present in the cell lysates at high glucose condition (Figure 4(b)). IL-8 is one of the key inflammatory cytokines during wound healing process, and it is a potent autocrine proangiogenic molecule [21]. IL-8 is chronically upregulated in diabetes but its expression is impaired in the acute inflammatory phase following injury in diabetic patients [22]. Another study reported that hyperglycemic condition did not affect IL-8 expression in dermal microvascular endothelial cells [17]. 

**Figure 5:**
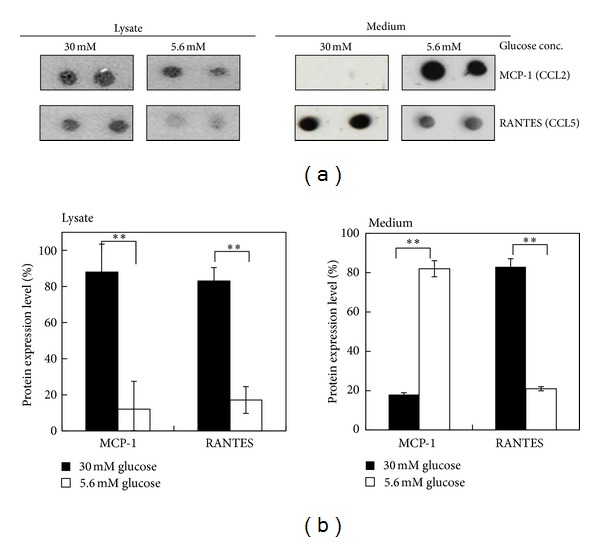
The effect of hyperglycemia on the expression of C-C motif chemokines in 3A-Sub-E trophoblast cells. 3A-Sub-E cells were exposed to 30 mM D-glucose or to 5.6 mM of D-glucose (control) for 48 h. Cell lysates (250–370 *μ*g) and the cultured conditioned media were collected for the antibody array analysis, respectively. (a) A representative dot image of the indicated C-C motif chemokines. (b) Average intensity for each pair of cytokine spots was quantitatively measured by Scion Image software. Each cytokine is represented by duplicate spots: MCP-1 (monocyte chemoattractant protein-1, CCL-2), RANTES (regulated upon activation, normal T-cell expression and secreted, CCL-5), and control (HRP-conjugated antibody as a positive control). Data are presented as mean ± SD (*n* = 4). **P < 0.01.

We also investigated C-C motif chemokine expressions.  Figure 5 showed that hyperglycemia induced significant expressions of regulated upon activation, normal T-cell expression and secreted (RANTES, CCL5). Monocyte chemo-attractant protein (MCP-1, CCL-2) was mainly expressed in the cell lysates under hyperglycemic condition and was present in the medium under normal glucose condition (Figure 5). MCP-1 plays a critical role in the recruitment and activation of leukocytes during acute inflammation. A study by deleting the expression of MCP-1 caused a significant upregulation of decorin, a small leucine rich proteoglycan (SLRPs) in acute wounds [23]. CCL-5 could upregulate decorin expression and its posttranslational modification [24]. Hyperglycemia elevated level of RANTES/CCL5 in patients with type 2 diabetes [25]. Inhibition of CCL2 and CCL5 may attenuate hyperglycemia and inflammation [26]. We previously have reported increased chondroitin sulfate proteoglycan, decorin, and biglycan expressions, and more chondroitin sulfate than heparin sulfate was substituted on perlecan core protein in the placenta with gestational diabetes mellitus (GDM) as well as in the trophoblasts cultured under hyperglycemic condition [11]. Heparin or heparan sulfate rather than chondroitin sulfate is involved in trophoblast implantation on uterine epithelium [27]. The present study suggested that our previous findings on hyperglycemia induced increased expressions of decorin and biglycan may be mediated by increased CCL-2 and CCL-5 expressions. In addition, C-C motif chemokines might also regulate GAG composition on perlecan. Hyperglycemia results in impaired placental structure, vascular abnormality, and embryo implantation defect during pregnancy.

**Figure 6:**
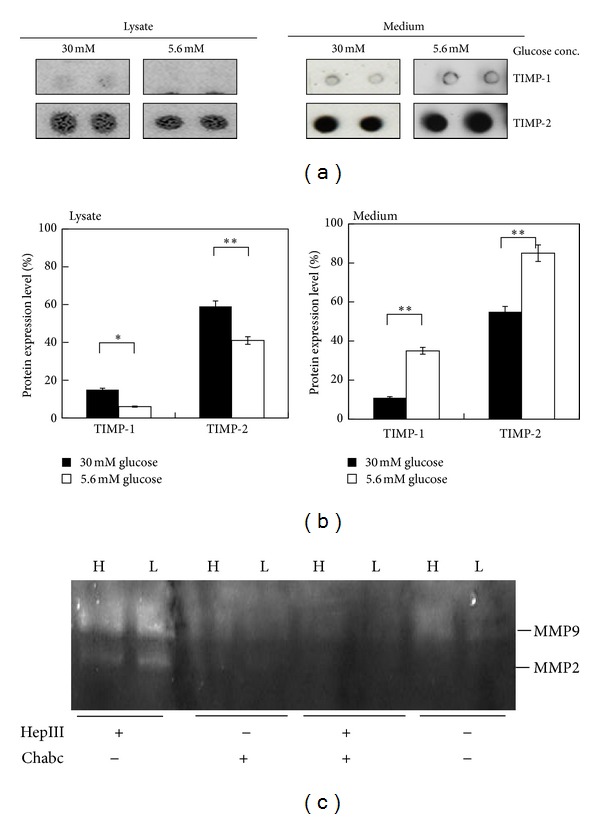
The effect of hyperglycemia on the expression of MMP inhibitors in 3A-Sub-E trophoblast cells. 3A-Sub-E cells were exposed to 30 mM D-glucose or to 5.6 mM of D-glucose (control) for 48 h. Cell lysates (250–370 *μ*g) and the cultured conditioned media were collected for the antibody array analysis, respectively. (a) Representative dot image of the indicated MMP inhibitor. (b) Average intensity for each pair of cytokine spots was quantitatively measured by Scion Image software. Data are presented as mean ± SD (*n* = 4). (c) A gelatin zymography gel image for MMP2 and MMP 9 activity. *P < 0.05. **P < 0.01. Each cytokine is represented by duplicate spots as indicated, TIMP-1 (tissue inhibitors of metalloproteinase-1), TIMP-2 (tissue inhibitors of metalloproteinase-2), and control (HRP-conjugated antibody as a positive control).

Placental development requires proper trophoblast invasion and tissue remodeling that involves the balanced matrix metalloproteinase (MMP) and their inhibitor, tissue inhibitor for MMP (TIMP) expressions. Figures 6(a) and 6(b) revealed that TIMP-2 is the major inhibitor expressed by trophoblasts. However, less TIMP-2 was present in the medium of 3A-Sub-E cultured under high glucose culture condition. Zymography analyses revealed that the expressions and activity of MMP2 and MMP9 were indeed increased in the cells with high glucose treatment (Figure 6(c)). Interestingly, the hyperglycemia induced MMP2 and MMP9 activities were diminished while 3A-Sub-E cells were treated with chondroitinase ABC. Our previous studies identified hyperglycemia-induced chondroitin sulfate proteoglycan expressions, such as decorin and biglycan, and more chondroitin sulfate content than heparin sulfate GAGs was substituted on perlecan core protein in 3A-Sub-E cells [11]. The present studies suggest that hyperglycemia resulting matrix remodeling also affects the secretion and activation of MMP and TIMP expressions by trophoblasts. Our findings are consistent with the other reports that induced hyperglycemic condition or diabetes increased MMP and decreased TIMP expressions [28, 29]. Trophoblasts are sensitive to the local microenvironment in responses to hyperglycemic condition and the extracellular matrix (ECM) composition and structure. 

### 3.4. The Effect of Hyperglycemia on the Expression of Perlecan Binding Growth Factors in Trophoblasts

**Figure 7:**
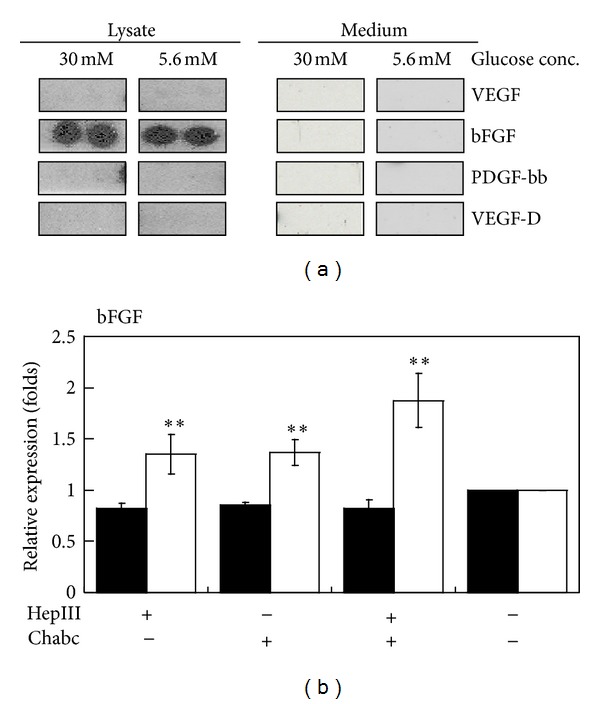
The effect of hyperglycemia on the expression of perlecan binding growth factors in 3A-Sub-E trophoblast cells. 3A-Sub-E cells were exposed to 30 mM D-glucose or to 5.6 mM of D-glucose (control) for 48 h. Cell lysates (250–370 *μ*g) and the cultured conditioned media were collected for the antibody array analysis, respectively. (a) A representative dot image of the indicated growth factors was shown. Compared to the control, only bFGF was expressed in the cell lysates. bFGF, basic fibroblast growth factor; VEGF, vascular endothelial growth factor; PDGF-bb, platelet derived growth factor-bb; VEGF-D, vascular endothelial growth factor D. (b) The mRNA expressions of bFGF in responses to heparinase (HepIII), chondroitinase ABC (Chabc), or both treatment in 3A-Sub-E cells under high (black bar) or normal glucose (white bar) culture condition. Compared to the control cells without any treatment at normal glucose condition, the mRNA level of bFGF was significantly increased in 3A-Sub-E cells cultured with HepIII, Chabc, or both treatments.

Perlecan can bind to proangiogenic growth factors, such as fibroblast growth factors (FGFs) and vascular endothelial growth factor (VEGF), and present them to their receptors on the cell surface [3, 4].  Figure 7(a) showed that basic fibroblast growth factor (bFGF) was present in cell lysates but not in the media, and VEGF, VEGF-D, or PDGF-bb was at nondetectable level in 3A-Sub-E cells. A recent report has confirmed that PDGF-bb is not derived from trophoblast [30]. Although an immunohistochemistry study revealed strong staining for VEGF and VEGFR-2 in vascular and trophoblastic cells from women with mild hyperglycemia and the staining for VEGFR-1 was discrete and limited to the trophoblast [31], we did not detect VEGF signal in the cultured 3A-sub-E trophoblasts. In addition, at normal glucose condition, the expression of bFGF is regulated by GAG composition in the extracellular matrix. Hyperglycemia-induced alterations of proteoglycan expressions and GAG compositions may impair the regulatory activity of HSPG such as perlecan on the regulations of bFGF (Figure 7(b)). Perlecan positively regulates angiogenesis by promoting bFGF binding to bFGF receptor which is heparin sulfate-dependent [4, 32–34]. 

### 3.5. Glycosaminoglycan Composition Substituted on the Proteoglycans Mediate Hyperglycemia-Induced Proinflammatory Cytokine and the Chemokine Expressions

**Figure 8:**
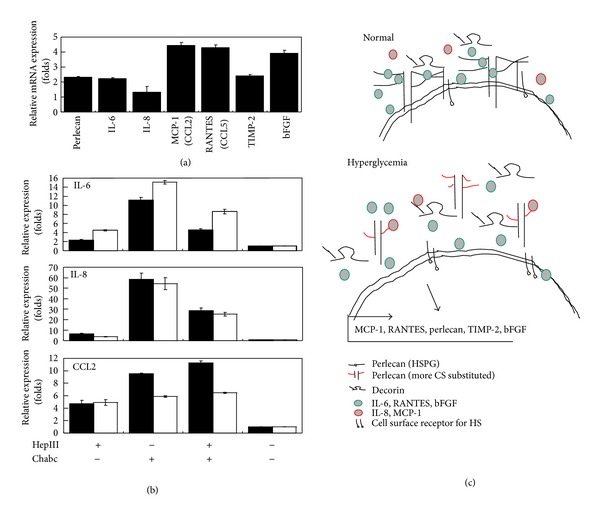
The effect of hyperglycemia induced altered glycosylation on the expressions of proinflammatory and chemokines in 3A-Sub-E trophoblast cells. Real-time quantitative polymerase chain reaction (RT-PCR) was performed to quantitatively measure. (a) The mRNA level of the indicated genes in responses to high glucose culture condition was increased at least 2 folds except IL-8 expression in 3A-Sub-E cells. (b) The mRNA expressions of IL-6, IL-8, and MCP-1 (CCL2) in 3A-Sub-E cells, were exposed to 30 mM D-glucose (black bar) or to 5.6 mM of D-glucose (normal control, grey bar) for 24 h followed by GAG degradation enzyme treatment with heparanase III (HepIII), chondroitinase ABC (Chabc), or both. Significantly increased expressions of IL-6, IL-8, and MCP-1 were occurred while 3A-Sub-E cells with Chondroitinase ABC treatment. (c) A diagram describes hyperglycemia-induced alterations of GAG substitution on perlecan (shorter and more chondroitin sulfate (CS) substituted than heparan sulfate (HS)) and matrix degradation by decreased TIMP-2 and increased activities of MMP2 and MMP9 resulting in the release of IL-6, RANTES, and bFGF into the extracellular matrix (soluble form) and enhance deposition of IL-8 and MCP-1 on the cell surfaces. The altered extracellular environment and the cell-associated and the soluble cytokine may contribute to the expressions of the indicated cytokines and chondroitin sulfate proteoglycans such as decorin at transcriptional and translational levels.

Previous studies have demonstrated that hyperglycemic condition indeed regulated the expressions of angiogenesis associated cytokines, MMPs, and growth factors at protein level. Real-time PCR studies confirmed the expressions of hyperglycemia sensitive cytokines at the mRNA level in 3A-Sub-E trophoblasts (Figure 8(a)). To further investigate whether the GAG composition may mediate hyperglycemic effect on the expressions of cytokines, GAG degradation enzyme treatment using heparanase III (HepIII), chondroitinase ABC (Chabc), or both in 3A-Sub-E cells cultured at high glucose condition was carried out to investigating the hyperglycemia sensitive cytokines at the mRNA level. Results indicated that treatment for eliminating heparin sulfate or/and chondroitin sulfate significantly increased mRNA expressions of IL-6, IL-8, and MCP-1 in the trophoblasts (Figure 8(b)) but did not affect the expressions of RANTES, TIMP-2 (data not shown), and bFGF (Figure 7(b)). It suggests that the hyperglycemia-induced expressions of IL-6, IL-8, and MCP-1 maybe mediated by the GAG composition and structure on the cell surface and the ECM. The regulatory mechanisms of heparin sulfate and chondroitin sulfate on the inflammatory cytokines maybe different. Heparan sulfate is essential for inflammatory response [35, 36]. The heparin binding interaction may retain IL-6, IL-8, and MCP-1 close to its sites of secretion that may favor a paracrine mode of activity [37, 38]. However, the soluble form of heparin sulfate may diminish the expression and production of inflammatory cytokines [39]. Chondroitin sulfate proteoglycans mainly present in the extracellular matrix may retain the cytokines such as IL-6 in the extracellular matrix and protect for further inflammation [40]. In addition, chondroitin sulfate as well as heparin sulfate function in regulation of inflammation may depen on their structure, size, and sequence [38]. Data in the present studies revealed that IL-8 and MCP-1 were mainly cell associated in trophoblasts at hyperglycemic condition and were present in the medium at normal glucose condition (Figures 4 and 5). It was known that induced hyperglycemia altered GAG substitution with more CS than HS on cell surface was not favorable for the binding with cytokines or growth factors. The increased cell-associated IL-8 and MCP-1 under hyperglycemic condition may be mediated by differ mechanism. These cytokines may play important roles on the regulation of inflammation and proliferation of 3A-Sub-E cells. The altered proteoglycan expression may result in the release of cytokines from the cell surface that may associate with increased levels of circulated inflammation related and angiogenesis associated cytokines in serum of women with GDM [41] or in patients with DM [42]. A diagram summarizing the possible regulatory mechanism of the altered glycosylation of proteoglycan on the expression of angiogenesis associated cytokines in trophoblast under hyperglycemic condition was shown in Figure 8(c). 

## 4. Conclusions

This study identified hyperglycemia-induced angiogenesis associated cytokine expressions by trophoblasts 3A-Sub-E. In addition, the altered proteoglycan expression and their glycosaminoglycan structure may regulate the activity of the angiogenesis associated cytokines and the matrix composition. Hyperglycemia-induced alterations in the GAG structure of proteoglycans and the angiogenesis cytokines may affect the growth and migration of trophoblasts that may contribute to the aberrant placental structure and the maternal and fetal complications during development.

## Figures and Tables

**Table 1:** Primer pairs for real-time quantitative polymerase chain reaction.

Target gene	Primer sequences
IL-6	F-AAATGCCAGCCTGCTGACGAAC
	R-AACAACAATCTGAGGTGCCCATGCTAC
IL-8	F-AACTTCTCCACAACCCTCTG
	R-TTGGCAGCCTTCCTGATTTC
MCP-1 (CCL-2)	F-CAGCCAGATGCAATCAATGCC
	R-TGGAATCCTGAACCCACTTCT
RANTES (CCL-5)	F-CCTCATTGCTACTGCCCTC
	R-CACTGGTGTAGAAATACTCC
TIMP-2	F-GCACATCACCCTCTGTGA
	R-CTCTGTGACCCAGTCCATCC
bFGF	F-GGCGTGTACATGTGGTCTCAGA
	R-TTATGGCTCACTGCAACCTTGA
b-actin	F-CATGTACGTTGCTATCCAGGC
	R-CTCCTTAATGTCACGCACGAT
